# The level of transforming growth factor-β as a possible predictor of cyclophosphamide response in children with steroid-resistant nephrotic syndrome

**DOI:** 10.37796/2211-8039.1205

**Published:** 2021-09-01

**Authors:** Ahmedz Widiasta, Kurnia Wahyudi, Yunia Sribudiani, Dedi Rachmadi

**Affiliations:** a Pediatric Nephrology Division, Child Health Department, Hasan Sadikin General Hospital, Faculty of Medicine, Universitas Padjadjaran, Bandung, Indonesia; b Epidemiology and Biostatistics Division, Public Health Department, Faculty of Medicine, Universitas Padjadjaran, Bandung, Indonesia; c Department of Biomedical Sciences, Division of Biochemistry and Molecular Biology, Faculty of Medicine, Universitas Padjadjaran, Indonesia

**Keywords:** Albumin-creatinin ratio, Correlation, Cyclophosphamide, Steroid-resistant nephrotic syndrome, TGF-β

## Abstract

**Background:**

Steroid-resistant nephrotic syndrome (SRNS) is a burden in the country due to the progressive severity of chronic kidney disease (CKD). Calcineurin inhibitors (CNIs) or monoclonal antibodies are currently recommended for the treatment of this disease. In developing countries, steroid and cyclophosphamide (CPA) are available drugs used during the treatment. This study aims to provide a non-invasive modality that can be used to predict the response of SRNS children to CPA therapy. Subsequently, the proteinuria duration was shortened to reduce the risk of glomerular damage. The present study aims to determine whether there is a correlation between baseline serum TGFB and proteinuria in SRNS children six months after receiving CPA treatment. The author hypothesized that there would be a negative correlation between those variables.

**Method:**

A prospective-cohort-study was conducted at Hasan Sadikin General Hospital Bandung, Indonesia. A total of 88 SRNS children, aged 1 to 18 were accessed for serum TGF-β level before receiving CPA therapy for six months, and clinical signs were observed. Furthermore, after six months of CPA treatment, the subjects were divided into CPA responder and non-responder based on the presence of proteinuria, then the data were analyzed using multiple logistic regression to adjust age and gender.

**Results:**

There was a statistically significant relationship between TGF-β and the risk of non-response to CPA therapy, after accounting for age, gender, baseline GFR, baseline ureum, and baseline urinary protein, the adjusted-OR was 1.051 (95% CI 1.007, 1.097, p = 0.022).

**Conclusion:**

The high level of serum TGF-β obtained prior to CPA administration are reliable data for estimating adverse results on CPA therapy. Based on these results, a high baseline TGF-β level correlates with the poor response of CPA therapy.

## 1. Introduction

Nephrotic syndrome is the most common glomerular disease discovered in children, and this entity is divided into two categories based on its steroid response, including steroid-resistant nephrotic syndrome (SRNS) and steroid-sensitive nephrotic syndrome (SSNS). The epidemiology of SRNS is approximately 10 – 20% of all NS [1,2], while FSGS is the predominant histopathologic finding associated with steroid resistance. Furthermore, SRNS is associated to an increased risk of end-stage renal disease (ESRD) development. It is also the main contributor to chronic kidney disease in children worldwide, while SRNS patients account for 15% of all children affected with chronic kidney disease (CKD) requiring renal-replacement therapy (RRT) [2–4]. According to the US Centers for Disease Control and Prevention (CDC), 37 million people in the United States, or approximately 15% of adults are estimated to have CKD. Of note, 90% of adults with CKD do not know they have it, one in two people with very low kidney function who are not on dialysis are not aware of the fact that they have CKD [5]. Similarly, SRNS with persistent proteinuria diagnosis within five years potentially developed into CKD stage V or end-stage kidney disease (ESKD) [6]. Similarly, persistent proteinuria is associated with poor quality of life reported by patients as a result of CKD, thromboembolic events, hypertension, peritonitis, persistent dyslipidemia, steroid toxicity, and death [6]. However, CKD is a non-communicable disease (NCD) that greatly increases children's mortality, decreases quality of life, eventually leading to kidney fibrosis, which is the final progressive kidney disease. Some evidence has shown that massive long-term protein loss leads to increased protein production in the extracellular matrix (ECM), decreased matrix breakdown, deregulation of cell matrix interaction, and inflammatory cell infiltration.

Low socioeconomic and developing countries have limited restrictions on managing SRNS and CKD, because not all developing countries have well-established national health insurance. Treatment of SRNS by the Kidney Disease Initiative Global Outcome (KDIGO) consists of CNIs or monoclonal antibodies, and an alkylating agent (CPA or chlorambucil) is not recommended due to poor outcome [6]. However, some developing countries only have CPA as an agent that is covered by their national health insurance. This condition requires previous CPA response prediction, for the healthy administration of CPA to SRNS children. According to the authors, there is a lack of well-published non-invasive biomarkers that predicts the SRNS response to CPA. A kidney biopsy is the recommended diagnostic tool to predict and assess response to SRNS treatment. Therefore, the serum TGF-β test is an alternative, less invasive diagnostic method for evaluating SRNS treatment response [6]. Kronbichler et al. (2016) showed the role of TGF-β1 in FSGS, especially in patients with a corticosteroid-resistant disease that develops into ESKD [7].

Massive proteinuria enhances the biological reaction and produce an inflammatory mediator such as *platelet-derived growth factor* (PDGF), endothelin-1, which is regulated upon activation of normal T-cell expressed and secreted (RANTES), monocyte chemoattractant protein-1 (MCP-1), osteopontin, tissue inhibitor of metalloproteinase-1 (TIMP-1), plasminogen activator inhibitor-1 (PAI-1), and transforming growth factor-beta (TGF-β) [8,9]. Therefore, a significant release results in overproduction of the extracellular matrix (ECM) and interstitial proliferation triggering glomerulus and tubulointerstitial fibrosis [8,9]. Transforming growth factor-beta 1 (TGF-β1) is the major fibrogenic growth factor that plays an important role in kidney scarring. Proteinuria is an indicator of a poor prognosis for various types of glomerular diseases and its toxic effect is related to the activation of tubular epithelial cells in the direction of increased production of cytokines and chemoattractant peptides [2,10,11]. TGF-β was produced as a result of proteinuria in tubular cells. In addition, TGF-β accumulates in interstitial and plays a role in stimulating cell proliferation by cell-induced extracellular matrix (ECM) proliferation. Secondarily, sclerosis and fibrosis are the product of ECM proliferation [12,13]. Studies on human kidney specimens confirmed that the three significant isoforms such as TGF-β1, TGF-β2, and TGF-β3 are expressed in the kidney [14–16]. Therefore, the baseline serum TGF-β1 level prior CPA therapy was examined to know the relationship between serum TGF-β level and the risk of non-response to CPA therapy. Based on the previous studies' theory, the authors hypothesized that SRNS with high TGF-β levels before CPA treatment would be those with extensive sclerosis and ongoing renal fibrotic processes. Therefore, they tend to experience CKD, which will reduce the quality of life and increase mortality. This study aimed to find a correlation between TGF-β before CPA treatment and proteinuria at six months after completing therapy.

## 2. Methods

The research, conducted in Hasan Sadikin General Hospital Bandung, Indonesia, September 2019 – February 2020, is a single based, prospective cohort analysis, which was approved by the Research Ethics Board of the University of Padjadjaran Medical School. The children diagnosed as SRNS by pediatric nephrologists were treated with CPA, and the TGF-β serum level was measured before undergoing CPA therapy. The SRNS is defined as failure in remission after four weeks of steroid administration [17]. The therapy was administered for six months, and clinical signs were observed because administration over a long period of time comes with a very high risk of undesirable side effects, especially for the gonads [1]. The observation requires intravenous administration of albumin, complications during treatment, and proteinuria. In the case of severe infection, autoimmune disease was omitted. Furthermore, after six months of CPA treatment, the observed result was protein loss, which was further divided into CPA responder and non-responder. CPA non-responders was defined as the group that have not achieved remission, after six-months of therapy with the used of this agent [1]. The kidney function with the Schwartz formula based on serum creatinine level was also observed.

### 2.1. Patients and methods

Serum was collected from 88 SRNS children, 31 with FSGS, 8 with MPGN, 1 with Mes-GN, and 13 with minimal change disease (MCD), and the TGF-β serum was measured by using Enzyme-linked immunosorbent assay (ELISA) method. Furthermore, laboratory data, including urinalysis, 24-h protein excretion, and creatinine clearance were collected from each patient. Baseline creatinine clearance was average in all patients. At the time of urine collection, patients with MCD and FSGS were receiving steroids.

### 2.2. Statistical analysis

The numerical data for baseline characteristics were presented using the mean (standard deviation) or median (min, max) appropriately. Categorical data were presented according to frequency and percentage. Multiple logistic regression was performed to analyze the relationship between the result variable CPA responder vs non-responder, coded as 0 and 1, and baseline TGF-β level, adjusting for age and gender. The adjusted odds ratio and 95% confidence interval were also presented, and all statistical tests were carried out with a significance level of 5%.

## 3. Results

The subjects were mostly male (62.5%), and had good kidney function based on their serum creatinine levels (Table 1). Most of the children in the non-responder group, 23.86% had focal-segmental glomerulosclerosis (FSGS) based on the kidney biopsy results, while the most common histopathological feature of SRNS in CPA responder group 14.77% was minimal-change disease (MCD) (Table 2). No child in the CPA responder group experienced decreasing glomerular filtration rate (GFR) based on the creatinine level of 88 SRNS children as shown in Table 3. Two out of the 39 children in the non-responder CPA group experienced decreasing GFR as shown in Table 4. 39 or 44.31% from 88 SRNS did not respond to six month CPA therapy as shown in Table 4.

This study showed a low average of TGF-β level in subjects that responded to CPA therapy after six months, with good kidney function of all the patients in Table 3. The high average level of TGF-β was reported in subjects that do not respond to CPA therapy, a decreased kidney function was also reported in 2 or 5.13% subjects (Table 4).

It was observed that there is a relationship between baseline serum TGF-β level and the CPA response among SRNS children after considering age, gender, baseline ureum level, and severity of urinary protein loss. For baseline characteristics, the numerical data using mean (standard deviation) or median (min, max), was presented appropriately. Furthermore, categorical data were presented according to frequency and percentage. Also, multiple logistic regression was also performed to determine the ratio of responder to CPA outcome variables vs. non-responder, coded as 0 and 1, and baseline TGF-β level, adjusting for age and gender. The Adjusted-odds ratio and 95% confidence interval were also presented, and statistical tests were performed using the 5% level of significance (Table 5). This study also analyzed whether baseline serum TGF-β level could be a predictor of SRNS histologically, i.e. FSGS or non-FSGS with the Mann-Whitney test. Unfortunately, of the 88 samples in this study, only 58 subjects obtained consent for kidney biopsy.

## 4. Discussion

Massive proteinuria is an enigma as it is a progressive CKD, with a complex mechanism [7,18,19], which increases the mortality rate of children and reduces quality of life [2,20]. Furthermore, steroid-resistant nephrotic syndrome in children is harmful, with almost half receiving CKD progression despite appropriate therapy [21–23]. Kidney fibrosis and matrix deposition are the typical long term effect of proteinuria [24,25]. The main proinflammatory factor in kidney fibrosis associated with proteinuria is TGF-β [2]. The pediatric nephrologist should correct the proteinuria as soon as possible, but certain conditions are different in each country. Although, every country has its unique condition, and some developing countries have some limitations in drug availability associated with their low-socioeconomic state or government policy. In addition, KDIGO recommends a guideline for performing kidney biopsy in children with SRNS, citizens of some countries tend to avoid invasive procedures such as kidney biopsy [26,27]. The result of the kidney biopsy is needed to evaluate the outcome of SRNS therapy, perhaps alternative drugs from other classes are needed. Such conditions allow the prediction of trends in their glomerulus to become fibrosis or an excessively matrix deposition.

All 88 SRNS children were treated with CPA based on its availability, and the SRNS children treated with calcineurin inhibitors (CNIs) were not enrolled, ie. Cyclosporine A or Tacrolimus. Most of the subjects were male, with a normal glomerular filtration rate (GFR), reflected from serum ureum and creatinine level (Table 1). Chemical formula of urea is (NH_2_)_2_ >C=O i.e. molecular weight of ureum is 60 while nitrogen is 28. 60/28 = 2.14, the conversion is blood urea nitrogen × 2.14 = blood urea, a calculative value. This was in accordance to the common etiology of the INS and secondary SRNS, mostly male. However, most of INS was responsive to steroid therapy. It has been suggested that some children were not adequately treated because prolonged neglected proteinuria led to certain sclerotic and fibrous glomeruli, which clinically manifested as FSGS [8,28,29]. This was also supported by the results of kidney biopsy, whereas FSGS were dominant in responders and non-responders group, and all of the MCD were responders (Table 2). Out of the 88 children that enrolled in this study, 39 children, or almost half have not responded well to CPA (Tabel 3), and it shows that almost half of SRNS in the population potentially become CKD. Furthermore, despite certain restrictions, the appropriate therapy for the treatment of SRNS is required. In persistent proteinuria, TGF-β is one of the main proinflammatory mediators, and the level predicts the treatment outcome. The longer the proteinuria lasts, the greater the likelihood that the kidney will have some irreversible entity like fibrosis or global sclerosis, which is the unfortunate outcome of CPA therapy. In addition, it was discovered that, despite the CPA therapy, almost half of the SRNS children had no remission, and this condition was predicted by a high level of baseline TGF-β, measured before CPA therapy (Table 4).

The nephrotic syndrome mostly undergoes remission with steroid treatment, and it is called idiopathic nephrotic syndrome (INS). Meanwhile, some relapse INS are not sensitive to steroid therapy, and there are known as secondary SRNS. Also, the non-responsiveness correlated with an increase in extracellular matrix (ECM) protein production, a decrease in matrix degradation, dysregulation of cell-matrix interaction, inflammatory cell infiltration, and transformation of resident cells [30]. Similarly, CKD's secondary SRNS pathogenesis is caused by the persistent proteinuria leading to inflammatory processes involving TGF-β, Vascular-endothelial growth factor (VEGF), platelet-derived growth factor (PDGF), and angiotensin (AT). TGF-β triggers the SMAD pathway that increases p21 and p15 as the beginning of the fibrotic kidney process leads to failure in the growth of glomerular and apoptosis. Persistent proteinuria also plays an important role in the glomerular cytoskeletal-protein defect that undergoes a relative-podocyte loss associated with hyperfiltration. This condition aggravates the previous inflammatory process, like a snow-ball phenomenon, and the severity of the disease or the onset of proteinuria in SRNS was estimated when TGF-β is circulated. In a combination of high serum TGF-β finding with the long-lasting proteinuria period, it is suggested that the fibrotic process has occurred, then it would be nearly impossible to be treated with an alkylating agent (CPA), because of its low remission rate [31]. Otherwise, alkylating agents could be used in a low serum combine with short-lasting proteinuria when the CNIs or monoclonal antibodies are not available. This limitation condition often occurs in poor countries, even in middle income countries.

This research is in line with a previous study conducted by Trihono et al. (2019), which reported that urinary excretion of TGF-β1 in children with the relapsing-nephrotic syndrome was as high as its level in SRNS. Significantly, it was much higher compared to the levels in nephrotic syndrome remission and those in children without kidney disease [2]. The study conducted by Wasilewska et al. (2004) also reported that the TGF-β1 level was the highest is SRNS before cyclosporine A therapy and reduced after three to twelve months of therapy, respectively [32]. Murakami et al. (1997) also reported that urinary TGF-β levels in the urine of IgA nephritis and FSGS were significantly higher compared to controls and patients with other forms of glomerular diseases, and significantly correlated with the grade of interstitial fibrosis [12].

TGF-β is a pleiotropic cytokine that is involved in kidney disease progression. An in vivo experiment has shown that the overproduction of TGF-β by mesangial cells, tubular epithelial cells, interstitial fibroblasts and interstitial fibroblasts, and macrophages [33]. Consistently, inhibition of TGF-β by anti-TGF-β antibody attenuated fibrosis in animal models of kidney disease [34–36]. In this study, a serum sample was used, due to some reasons, the urine or tissue sample was excluded. First, TGF-β can be discovered not only in the kidney and urine, but also in the blood. There are no studies linking serum TGF-β with the degree of proteinuria in SRNS yet. Whereas, this is very important as SRNS therapy with persistent proteinuria is primarily aimed at preventing or at least delaying the progression of chronic kidney disease (CKD) to end-stage kidney disease (ESKD). Some of the developing novel therapies are those using TGF-β as a target. Therefore, the serum level of TGF-β in SRNS patients with various degrees of proteinuria is absolutely needed. Previous established studies have linked SRNS to TGF-β in the urine and kidney tissue. CPA administered intravenously, its effects through the alkylation of deoxyribonucleic acid (DNA) [37–39], this process can also influence the expression of the TGFB1, TGFB2, and TGFB3 genes. Due to its circulatory status in the body, there are several current developing SRNS agents such as monoclonal antibodies (fresolimumab), anti-microRNA (antimir), and quercetin using TGF-β as a therapeutic target [40–42]. Therefore, the profile of TGF-β level from serum would absolutely essential, in addition, these drugs are usually given intravenously, and there will be many effects on TGF-beta in the circulation and other organs.

The present study is the first to associate remission with serum TGF-β before alkylating agent therapy, with prospective-cohort design. This was concluded from the multiple regression analysis with a statistically significant relationship between TGF-β and the risk of non-response to CPA therapy, after accounting for age and gender. Multiple logistic regression was performed to determine the relationship between the responder and the CPA outcome variable vs. non-responder, coded as 0 and 1, and baseline TGF-β level, adjusting for age and gender. The adjusted-odds ratio and its 95% confidence interval were also presented. All statistical tests were performed using the level of significance of 5%, and the power of this study is statistically excellent (Table 5). Unfortunately, this study had several limitations, i.e, it could not link TGF-β with kidney histopathology, as some subjects refused to undergo kidney biopsy.

This result showed that it would be preferable not to use CPA in a situation where elevated baseline TGF-β levels are reported in SRNS children. In some developing countries, it was nearly impossible to use a monoclonal antibody at the first SRNS diagnosis. Meanwhile, CNIs are more reliable, it was reported that the cost of the CNIs price is higher than that of the alkylating agent. Therefore, some national insurance often limits CNIs and prefer the use of an alkylating agent, due to the cost.

## 5. Conclusion

TGF-β plays an important role in the treatment of SRNS in developing countries where repeated kidney biopsy is limited. Further study is needed to confirm the role of baseline TGF-β level as prognostic factor of CPA response of SRNS children. Meanwhile, it was discovered that an alkylating agent such as CPA is not the best treatment for SRNS, but it is used in developing countries due to its availability and some economic reason. Therefore, it is recommended that CPA be avoided when using SRNS for children with high baseline TGF-β levels.

## Figures and Tables

**Table 1:** Baseline characteristics of the study participants.

Variables	n = 88
Age (years), mean (SD)	8.8 (3.9)
Gender, frequency (%)	
Female	33 (37.5)
Male	55 (62.5)
Ureum (mg/dL), median (min, max)	47 (39, 88)
Creatinine (mg/dL), median (min, max)	0.8 (0.6, 3.9)
Urinary protein (g/24h), median (min, max)	2.0 (1.5, 3.5)
GFR, (mL/min/1.73 m^2^)	110 (22, 150)
Baseline TGF-β (pg/mL), median (min, max)	23.31 (12.15, 75.32)

**Table 2:** Kidney biopsy results.

Histopathologic findings, n (%)	CPA non-responder	CPA responder
FSGS	21 (23,86)	10 (11.36)
MPGN	5 (5,68)	3 (3,41)
Mes-GN	1 (1.14)	0
MCD	0	13 (14.77)
Refused biopsy	12 (13,64)	23 (26.14)

FSGS, Focal-segmental glomerulosclerosis; MPGN, Membranoproliferative glomerulonephritis.

Mes-GN, Mesangial-proliferative glomerulonephritis; MCD, Minimal change disease.

**Table 3:** Baseline TGF-β level, kidney function and urinary protein at baseline and after six months of therapy in CPA-responder group.

No.	Age (Year)	Gender	Laboratory measurement at initial cyclophosphamide therapy				Laboratory measurement in the end cyclophosphamide therapy		
	
			Ureum (mg/dL)	Creatinine (mg/dL)	Urinary protein (g/day)	TGF-β (pg/mL)	Ureum (mg/dL)	Creatinine (mg/dL)	Urinary protein (g/day)
1	8	M	39	0.7	1.5	15.03	35	0.7	0.2
2	10	M	42	0.7	1.9	17.07	40	0.7	0.2
3	2	M	45	0.6	1.5	14.69	43	0.7	0.3
4	6	F	48	0.8	1.9	23.65	44	0.7	0.1
5	6	M	49	0.7	2.1	21.94	46	0.7	0.2
6	7	F	45	0.8	2.0	18.45	45	0.8	0.1
7	11	M	47	0.9	1.7	27.98	44	0.7	0.2
8	4	M	49	0.6	1.9	18.23	47	0.7	0.1
9	12	M	44	0.7	2.3	15.74	45	0.7	0.3
10	8	M	49	0.8	2.2	29.34	47	0.8	0.2
11	7	F	47	0.6	3.5	26.86	44	0.8	0.2
12	10	F	42	0.7	2.0	22.63	47	0.7	0.2
13	11	M	49	0.6	2.0	20.38	46	0.7	0.1
14	10	M	46	0.7	2.0	15.2	47	0.8	0.2
15	1	F	45	0.6	2.0	24.12	45	0.7	0.1
16	8	M	43	0.7	2.0	25.3	44	0.7	0.2
17	10	M	45	0.7	1.5	20.91	43	0.8	0.2
18	11	M	47	0.8	3.4	17.37	46	0.8	0.2
19	4	M	44	0.7	2.5	35.64	43	0.7	0.2
20	4	M	48	0.8	2.0	18.72	44	0.8	0.2
21	4	M	50	0.7	2.5	27.4	47	0.7	0.2
22	11	M	51	0.8	2.0	19.93	47	0.8	0.2
23	9	M	55	0.7	2.1	18.43	45	0.7	0.1
24	8	F	49	0.6	2.0	59.38	44	0.7	0.2
25	10	M	48	0.9	2.0	20.22	45	0.9	0.2
26	9	F	47	0.7	2.0	17.84	43	0.7	0.2
27	13	F	48	0.8	2.1	26.48	46	0.8	0.2
28	12	M	49	0.6	2.0	12.15	46	0.7	0.1
29	7	M	46	0.7	1.9	14.71	46	0.7	0.2
30	17	M	44	0.8	1.7	42.28	47	0.8	0.2
31	10	M	45	0.7	1.6	22.3	45	0.7	0.2
32	10	F	43	0.7	1.7	41.24	48	0.7	0.2
33	17	F	44	0.8	3.4	13.43	47	0.8	0.1
34	14	M	47	0.7	1.8	18.3	44	0.7	0.2
35	5	M	48	0.6	2.0	19.54	47	0.7	0
36	8	M	44	0.7	2.0	28.47	44	0.7	0.2
37	11	M	48	0.8	3.0	45.98	47	0.8	0
38	11	F	45	0.7	3.1	52.71	44	0.7	0.1
39	9	F	47	0.8	2.2	22.96	48	0.8	0.2
40	9	M	47	0.7	2.0	16.84	47	0.7	0
41	4	M	48	0.6	2.0	19.98	45	0.6	0.1
42	7	M	49	0.7	1.9	12.22	46	0.7	0
43	17	M	45	0.8	1.8	27.85	47	0.8	0
44	12	M	43	0.7	1.9	27.47	47	0.7	0
45	9	F	47	0.7	2.0	21.75	46	0.7	0.1
46	3	M	50	0.8	2.0	14.02	48	0.8	0.1
47	5	M	45	0.8	2.0	14.74	48	0.8	0.1
48	6	F	48	0.7	2.0	13.34	48	0.7	0.1
49	5	M	49	0.7	2.0	48.33	47	0.7	0.1

**Table 4:** Baseline TGF-β level, kidney function and urinary protein at baseline and after six months of therapy in CPA-nonresponder group.

	Age (Year)	Gender	Laboratory measurement at initial cyclophosphamide therapy				Laboratory measurement in the end cyclophosphamide therapy		
	
			Ureum (mg/dL)	Creatinine (mg/dL)	Urinary protein (g/day)	TGF-β (pg/mL)	Ureum (mg/dL)	Creatinine (mg/dL)	Urinary protein (g/day)
1	12	M	88	1.7	3.5	44.43	101	2.1	3.5
2	10	M	45	0.8	2.0	35.48	46	0.8	1.0
3	9	M	49	0.7	2.5	18.02	48	0.7	1.5
4	10	F	41	0.7	2.5	17.42	45	0.7	1.5
5	7	M	50	0.9	2.0	46.51	46	0.8	1.5
6	7	M	47	0.8	2.5	35.13	48	0.8	1.5
7	11	M	43	0.6	2.5	22.63	44	0.7	1.5
8	8	M	45	0.7	2.5	18.91	46	0.7	1.5
9	8	M	49	0.7	2.5	30.49	47	0.7	1.5
10	7	M	42	0.6	2.3	17.29	45	0.6	2.0
11	7	F	47	0.7	2.0	51.88	46	0.6	1.5
12	1	F	49	0.8	2.0	25.71	47	0.7	1.5
13	12	F	42	0.7	2.5	28	45	0.7	1.5
14	8	F	46	0.6	2.0	13.72	45	0.6	1.5
15	14	M	44	0.8	2.0	21.68	46	0.7	1.5
16	14	F	41	0.7	2.1	24.36	45	0.8	1.5
17	4	F	49	0.7	2.5	20.41	47	0.8	1.5
18	4	M	50	0.6	2.5	28.62	47	0.7	1.5
19	5	F	44	0.7	2.5	22.6	46	0.9	2.0
20	2	F	48	0.6	2.0	20.8	47	0.8	1.5
21	13	F	48	0.7	2.0	23.65	47	0.8	1.5
22	13	F	42	0.7	2.1	19.11	44	0.9	1.5
23	8	M	45	0.8	2.1	30.72	46	0.8	1.5
24	8	M	48	0.9	2.0	51.82	47	0.9	1.5
25	17	F	47	1.0	3.0	21.08	47	0.9	2.0
26	14	M	45	3.9	3.5	29.35	47	5.1	3.5
27	8	M	42	0.9	2.0	29.12	46	0.9	1.5
28	4	F	44	0.8	2.1	49.41	46	0.8	1.5
29	13	M	49	0.7	2.0	51.48	47	0.7	1.5
30	4	F	51	0.8	2.0	23.92	46	0.8	1.5
31	10	F	50	0.7	2.0	39.44	47	0.8	1.5
32	14	M	47	0.8	2.1	27.63	48	0.7	1.5
33	7	F	49	0.7	1.9	43.97	47	0.7	1.0
34	14	F	44	0.8	2.0	44.18	46	0.8	1.5
35	2	F	48	0.7	2.3	39.16	45	0.7	1.5
36	5	F	49	0.6	2.0	36.26	46	0.6	1.5
37	14	M	45	0.8	3.5	75.32	47	0.7	1.5
38	15	M	44	0.7	2.5	28.11	47	0.7	1.5
39	11	M	49	0.7	2.0	20.75	48	0.8	1.5

**Table 5:** Multiple regression logistic.

Effect	β	SE	Wald	p	OR (95% CI)
Baseline TGF-β	0.050	0.022	5.257	0.022	1.051 (1.007, 1.097)
Age	0.012	0.063	0.034	0.853	1.012 (0.895, 1.144)
Gender (female vs male)	0.782	0.485	2.596	0.107	2.186 (0.844, 5.660)
Baseline GFR	−0.019	0.016	1.333	0.248	0.981 (0.951, 1.013)
Baseline ureum	0.001	0.055	0.001	0.982	1.001 (0.900, 1.114)
Baseline urinary protein	0.900	0.591	2.316	0.128	2.459 (0.772, 7.835)
Constant	−4.024	2.677	2.261	0.133	

TGF-β = Transforming growth factor-β; β = regression coefficient; SE = standard error; OR = Odds ratio; CI = confidence interval.
